# A chemoenzymatic process for amide bond formation by an adenylating enzyme-mediated mechanism

**DOI:** 10.1038/s41598-018-21408-8

**Published:** 2018-02-13

**Authors:** Ryotaro Hara, Kengo Hirai, Shin Suzuki, Kuniki Kino

**Affiliations:** 1 Research Institute for Science and Engineering, Waseda University, Tokyo, 169-8555 Japan; 2 Department of Applied Chemistry, Faculty of Science and Engineering, Waseda University, Tokyo, 169-8555 Japan

## Abstract

Amide bond formation serves as a fundamental reaction in chemistry, and is practically useful for the synthesis of peptides, food additives, and polymers. However, current methods for amide bond formation essentially generate wastes and suffer from poor atom economy under harsh conditions. To solve these issues, we demonstrated an alternative synthesis method for diverse tryptophyl-*N*-alkylamides by the combination of the first adenylation domain of tyrocidine synthetase 1 with primary or secondary amines as nucleophiles. Moreover, the physiological role of this domain is l-phenylalanine adenylation; however, we revealed that it displayed broad substrate flexibility from mono-substituted tryptophan analogues to even d-tryptophan. To the best of our knowledge, this is the first evidence for an adenylating enzyme-mediated direct amide bond formation via a sequential enzymatic activation of amino acids followed by nucleophilic substitution by general amines. These findings facilitate the design of a promising tool for biocatalytic straightforward amide bond formation with less side products.

## Introduction

Amide bonds broadly appear in a vast number of organic compounds, including both biologically active natural products and synthetic chemicals. Thus, amide bond formation can be an important step in the synthesis of pharmaceuticals, bioactive peptides, food additives, flavours, nutrients and polymers. In addition, amide bond formation plays a significant role in synthesising useful compounds for industrial applications^1^. Various methods for amide bond formation have been developed to date. Typical amide synthesis, such as solid-phase peptide synthesis (SPPS)^2^, depends on a condensation reagent^3^ to yield a highly reactive ester for reaction with amines, resulting in amide bond formation. Alternatively, amide bond is also formed by the acylation of amines with carboxylic acid chloride in the presence of a base (Schotten-Baumann reaction)^4^. These chemical methods are well established and would give good yields; however, they have some underlying drawbacks, such as harmful conditions, toxicity of reagents, and side product formation. SPPS also requires protection and deprotection procedures to avoid unnecessary side reactions, and considering the stoichiometric amount of the condensation reagent, it generates at least an equal amount of side product per product formed^1,3^. Hence, the requirement for a precisely controlled amide bond formation reaction with lesser steps and improved efficiency remains a serious concern for practical purposes. Catalytic methods like boron-based catalysis or metal-based catalysis, including titanium or zinc complex, are also potential methods for amide production^5^. However, these reactions need to be carried out under harsh conditions and they suffer from undesirable side reactions. Hence, an efficient, selective, mild, and environment-friendly strategy for a method independent of condensation reagent, which results in less side products, remains in high demand for amide bond formation reactions^4^.

In nature, the most abundant biological macromolecules linked with amide bonds are peptides and proteins synthesised by a ribosome system in living organisms. Biocatalytic amide bond formation is achieved by various enzymatic or chemoenzymatic methods, i.e. aminolysis reaction by hydrolases^6,7^, peptide formation between amino acid esters and amino acids by acyltransferases^8–10^, ligation with two or more unprotected amino acids by ATP-dependent ligases^11–16^, nucleophilic substitution by transpeptidases^17^, and hydration of nitrile to amide by nitrile hydratases^18^. A nonribosomal peptide synthetase (NRPS) is another such method commonly known as a ribosome-independent biological system for secondary metabolite production in bacteria and fungi, and they are composed of huge multidomain modular enzymes^19^. An adenylation domain (A domain), one of the essential region in NRPS involved in the first step of ATP-driven nonribosomal peptide synthesis, is critical for the selection and incorporation of a specific amino acid into a peptide scaffold, and it activates a carboxy group of the amino acid accompanied by hydrolysis of ATP to AMP and pyrophosphate to form the corresponding aminoacyl-AMP intermediate^20^.

The truncated A domain of tyrocidine synthetase 1 (TycA) produced in *Escherichia coli* catalyses the formations of the following dipeptides: l-phenylalanyl-l-phenylalanine, l-phenylalanyl-l-alanine, l-phenylalanyl-l-phenylalaninamide, and l-phenylalanyl-l-leucinamide^21^. However, this method is applicable only to the combination of amino acids (amide); hence, the products are limited to dipeptides. More recently, acyl-CoA synthetase AcsA from *Pseudomonas chlororaphis* B23 was identified to catalyse amide bond formation via a thioester bond between short chain fatty acids and l-cysteine^22^. Likewise, DltA is responsible for transferring d-alanine to a carrier protein, DltC in *Bacillus subtilis* 168, and it catalyses the synthesis of various dipeptides containing cysteine at C-terminal and some oligopeptides carrying d- or l-cysteine at the second N-terminal position^23^. In both these biocatalytic cases, thiol-based compounds play a key role in amide bond formation, because a nucleophilic attack is triggered by a thiol group followed by non-enzymatic *S-* to *N-* acyl transfer resulting in amide bond formation. To the best of our knowledge, little information is available concerning direct amidation yielding in amide bond formation between the acyl group of amino acids and general amines.

Here, for the identification of new adenylating enzyme-mediated amide bond formation methods, we have demonstrated l-tryptophyl-*N*-alkylamide synthesis by enzymatic adenylation of the carboxy group of l-tryptophan followed by nucleophilic substitution with diverse amines.

## Results

### l-Tryptophyl-*N*-alkylamides synthesis by TycA-A

We have demonstrated previously that the A domain of TycA (TycA-A) can be used for the synthesis of l-tryptophyl-l-proline^24^. This finding suggested that the carboxy group of l-tryptophan was first adenylated by TycA-A followed by nucleophilic substitution by the amino group of l-proline. Based on this phenomenon, we speculated that amide bond would be formed directly by activating the carboxy group of the amino acid using an A domain followed by nucleophilic substitution of general amines. This hypothetical reaction mechanism would enable the synthesis of a wide range of amides as well as peptides, and this system should serve as a versatile platform of enzyme-mediated amide bond formation. To investigate the nucleophile specificity of TycA-A, the following nucleophilic species were used in l-tryptophyl-*N*-alkylamide synthesis: methylamine, dimethylamine, trimethylamine, β-alanine, γ-aminobutyric acid, azetidine, pyrrolidine, piperidine, azepane, azocane, d-proline, *cis*-4-hydroxy-l-proline, *cis*-4-hydroxy-d-proline, l-prolinamide, and l-azetidine-2-carboxylic acid. After the reaction, HPLC analysis of the reaction mixture showed that specific peaks corresponding to newly formed products were detected in all amines except for trimethylamine. As speculated, an amide bond was probably formed by the enzymatic adenylation of l-tryptophan and subsequent nucleophilic substitution by amines. Each product was calculated from the amount of decrease in l-tryptophan minus the amount of l-tryptophyl-l-tryptophan generation, and the yield was calculated against the added l-tryptophan concentration (Table 1).

**Table 1:** MS detection of amide products formed by activation of l-tryptophan by TycA-A followed by nucleophilic substitution.

Substrate for TycA-A	Nucleophile	^*a*^Product (mM)	^*b*^Conversion (%)	Protonated parent ion (*m/z*)	MS/MS fragment ion (*m/z*)			
					^*c*^Type 1		^*d*^Type 2	
l-Tryptophan	*(Linear amines)*							
	Methylamine	0.81	8.1	218.1302	58.0300	159.0923	87.0552	130.0655
	Dimethylamine	0.81	8.1	232.0858	72.0446	159.0915	101.0705	130.0648
	β-Alanine	0.96	9.6	276.1371	116.0508	159.0930	145.0653	130.0674
	γ-Aminobutyric acid	1.16	11.6	290.1532	130.0663	159.0934	ND	ND
	*(Cyclic amines)*							
	Azetidine	0.82	8.2	244.1457	84.0450	159.0927	113.0713	130.0657
	Pyrrolidine	0.92	9.2	258.1301	98.0598	159.0916	127.0863	130.0646
	Piperidine	1.40	10.4	272.1783	112.0767	159.0930	141.1038	130.0661
	Azepane	0.52	5.2	286.1944	126.0927	159.0933	155.1198	130.0663
	Azocane	1.35	13.5	300.2090	140.1087	159.0936	169.1350	130.0665
	*(Cyclic amino acids and derivative)*							
	d-Proline	0.46	4.6	302.1566	142.0647	159.0917	171.0760	130.0647
	*cis*-4-Hydroxy-l-proline	0.94	9.4	318.1136	ND	159.0910	187.0707	130.0642
	*cis*-4-Hydroxy-d-proline	0.78	7.8	318.1481	ND	159.0934	187.0742	130.0662
	l-Prolinamide	0.85	8.5	301.1682	ND	159.0940	170.0626	130.0666
	l-Azetidine-2-carboxylic acid	0.78	7.8	288.1772	128.0342	159.0918	157.0604	130.0649

ND, not detected

^*a*^Calculated from the amount of decrease in l-tryptophan minus the amount of l-tryptophyl-l-tryptophan generation.

^*b*^Calculated against the added l-tryptophan concentration.

^*c*^Fragment ion generated by C_1_-C_2_ cleavage.

^*d*^Fragment ion generated by C_2_-C_3_ cleavage.

### Identification of l-tryptophyl-*N*-alkylamides by MS/MS and NMR spectra

**Figure 1:**
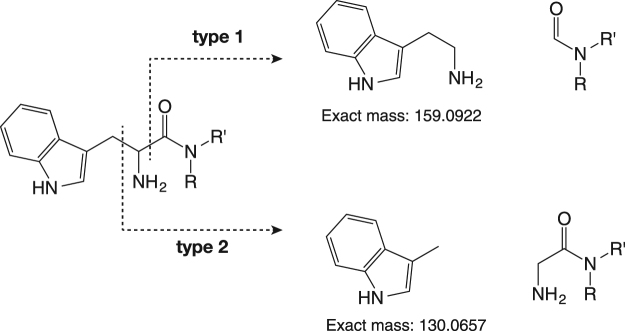
Fragmentation pattern of the reaction products.

The structures of various amides from l-tryptophan with amines were identified by MS/MS and NMR analyses. The amides produced in the reaction mixture were isolated by HPLC equipped with a fraction collector. Each mass-to-charge ratio (*m*/*z*) of protonated ions resulting from products was identical to that of the proposed structures for each l-tryptophyl-*N*-alkylamide. Further, these structures were identified by assignment of fragment ion peaks obtained from MS/MS spectra to the two cleavage fragments, as expected (Figs 1,S1). We have assigned the structures for various amides deduced from these MS/MS fragments (Table 1).

**Figure 2:**
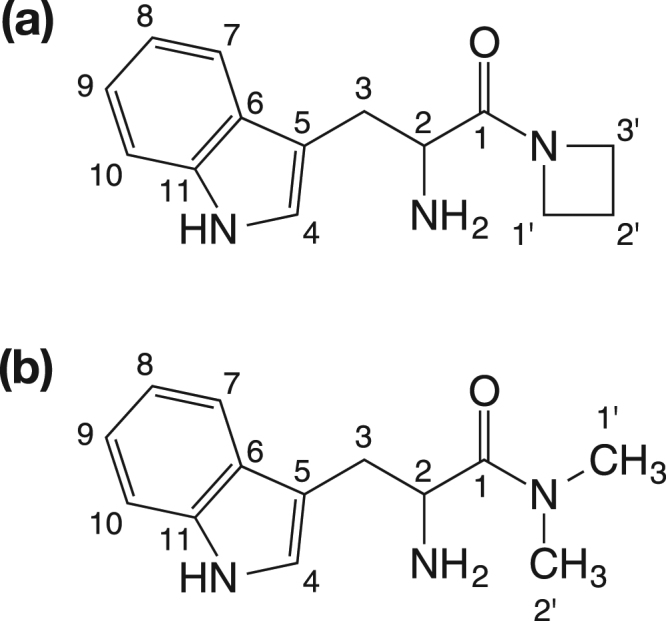
Structures of l-tryptophyl-*N*-alkylamide. (**a**) l-tryptophylazetidine. (**b**) l-tryptophyldimethylamine.

**Figure 3:**
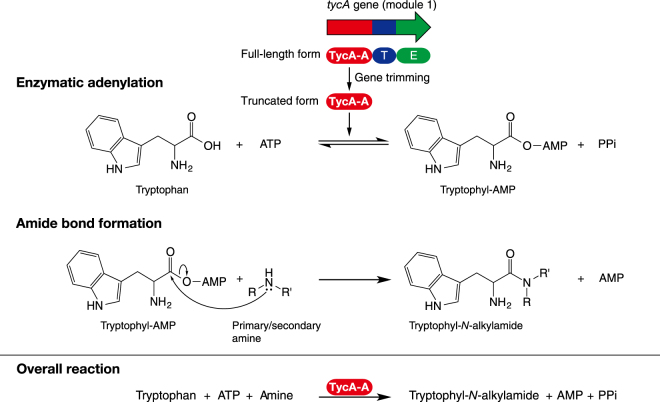
Schematic representation of chemoenzymatic amide bond formation using the truncated adenylating enzyme, TycA-A.

In addition, l-tryptophylazetidine as a typical cyclic amine and l-tryptophyldimethylamine as a typical linear amine were characterized by NMR (Tables 2,3 Figs S2,S3). The identified structures were in good agreement with the proposed structures as shown in Fig. 2. Consequently, it is uncovered that the nucleophilic substitution by amines had occurred to the carbonyl moiety of l-tryptophyl-AMP intermediate, and then an amide bond formed between l-tryptophan and various amines (Fig. 3).

**Table 2:** NMR spectrum data of l-tryptophylazetidine.

Position no.^*a*^	Chemical shift (δ ppm)		
	^13^C NMR	^1^H NMR (integration, multiplicity, *J* value [Hz])	
1	171.1	—	
2	53.1	4.20	(1H, dd, 9.0, 6.0)
3	29.3	3.36	(1H, dd, 14.4, 6.0)
		3.30	(1H, dd, 14.4, 9.0)
4	128.1	7.33	(1H, s)
5	109.5	—	
6	129.6	—	
7	120.9	7.60	(1H, ddd, 8.1, 0.9, 0.6)
8	125.2	7.21	(1H, ddd, 8.1, 7.2, 0.9)
9	122.6	7.29	(1H, ddd, 7.8, 7.2, 0.9)
10	115.0	7.55	(1H, ddd, 7.8, 0.9, 0.6)
11	139.1	—	
1′	51.6	3.94	(1H, tdd, 9.9, 6.0, 0.6)
		3.71	(1H, tdd, 9.9, 6.0, 1.2)
2′	17.7	2.07	(1H, dtt, 11.4, 9.6, 6.0)
		1.75	(1H, dtt, 11.4, 9.6, 6.0)
3′	50.6	4.02	(1H, tdd, 9.6, 6.0, 1.2)
		3.11	(1H, tdd, 9.6, 6.0, 0.6)

^*a*^Position numbers are shown in Fig. 2(a).

**Table 3:** NMR spectrum data of l-tryptophyldimethylamine.

Position no.^*a*^	Chemical shift (δ ppm)		
	^13^C NMR	^1^H NMR (integration, multiplicity, *J* value [Hz])	
1	172.1	—	
2	53.8	4.73	(1H, dd, 9.8, 6.0)
3	29.3	3.39	(1H, dd, 14.4, 6.0)
		3.34	(1H, dd, 14.4, 9.8)
4	128.2	7.31	(1H, s)
5	109.2	—	
6	129.6	—	
7	120.9	7.55	(1H, ddd, 7.8, 0.9, 0.6)
8	125.2	7.27	(1H, ddd, 7.8, 7.2, 0.9)
9	122.5	7.19	(1H, ddd, 8.1, 7.2, 0.9)
10	115.0	7.53	(1H, ddd, 8.1, 0.9, 0.6)
11	139.2	—	
1′	38.6	2.81	(3H, s)
2′	39.7	2.71	(3H, s)

^*a*^Position numbers are shown in Fig. 2(b).

### Substrate specificity of TycA-A

It was assumed that if TycA-A could accept l-tryptophan analogues, more diverse amides would be synthesised. To investigate the amide synthesis using other substrates, 6-fluorotryptophan, 6-chlorotryptophan, 6-bromotryptophan, and 5-hydroxytryptophan as l-tryptophan analogues were used in place of l-tryptophan. HPLC analysis of the reaction mixtures identified the presence of newly formed product peaks. The chromatograms of the reaction mixture containing 6-fluorotryptophan and amines gave the product peaks at 7.15 min, 5.71 min, and 5.98 min, corresponding to 6-fluorotryptophyl-l-proline, 6-fluorotryptophyldimethylamine, and 6-fluorotryptophylazetidine, respectively (Fig. S4). Similarly, the respective product peaks were observed in case of 6-chlorotryptophan, 6-bromotryptophan, and 5-hydroxytryptophan (Figs S5–S7). As summarized in Table 4, mono-substituted l-tryptophan analogues were also available for adenylation by TycA-A, and the presence of corresponding products could clearly be confirmed.

**Table 4:** MS analysis of the ligation products between tryptophan analogs with typical amines.

Substrate for TycA-A	Nucleophile		
	l-Proline	Azetidine	Dimethylamine
6-Fluorotryptophan	320.1475	262.1401	250.1401
6-Chlorotryptophan	336.1170	278.1109	266.1119
6-Bromotryptophan	380.0649	322.0614	310.0617
5-Hydroxytryptophan	318.1503	260.1415	248.1448

### Stoichiometry

**Figure 4:**
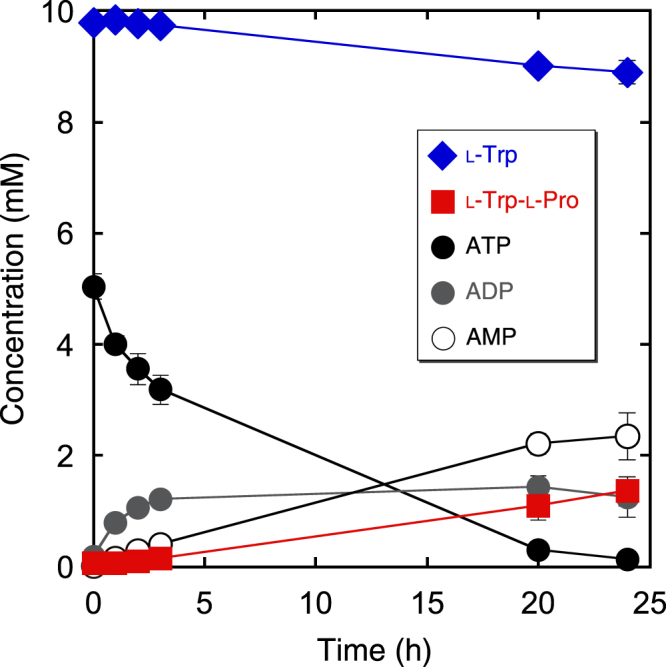
Time course of l-tryptophyl-l-proline synthesis using TycA-A.

Time course of l-tryptophan consumption and AMP formation during the synthesis was examined. Although 1.4 mM l-tryptophyl-l-proline was synthesised after a 24-h reaction, little correlation between l-tryptophyl-l-proline formation and AMP formation was found in this reaction (Fig. 4). The major reason for this discrepancy is possibly due to the side reactions. In fact, formation of a smaller amount of l-tryptophyl-l-tryptophan along with l-tryptophyl-l-proline and an unfavourable degradation of ATP to ADP were confirmed in the reaction mixture.

## Discussion

In this study, we have demonstrated the synthesis of various l-tryptophyl-*N*-alkylamides by a sequential process as per the following stepwise mechanism: (i) l-tryptophan was enzymatically adenylated by TycA-A to form an l-tryptophyl-AMP intermediate, and (ii) a nucleophilic substitution by an amine to l-tryptophyl-AMP led to an enzyme-independent amide bond formation (Fig. 3).

We have recently reported the synthesis of various aminoacyl-l-prolines, such as l-tryptophyl-l-proline, l-aspartyl-l-proline, and l-lysyl-l-proline using TycA-A, SurfB2-A, and BacB1-A, respectively^24^. These adenylating enzyme-catalysed reactions would be achieved by nucleophilic substitution by l-proline to aminoacyl-AMP intermediates. This could mean that if a nucleophilic substitution was actually carried out on an aminoacyl-AMP, an enzyme-independent amide bond formation would be established. We tested this in a reaction by using TycA-A as a model adenylating enzyme with various amines as nucleophiles. Formation of amides could be confirmed for all the reagents tested (Table 1). It is noted that trimethylamine, a tertiary amine, was inert because it was significantly less nucleophilic than primary or secondary amines. In general, tertiary amines are sterically hindered bases that are poor nucleophiles, and thereby it seemed to be hard to enhance the inherent reactivity of an amine in the chemoenzymatic method.

Interestingly, mono-substituted tryptophan analogues and even its d-isomer were accepted as substrates for TycA-A (Table 4), suggesting that TycA-A is a more promiscuous enzyme than previous reports suggested^25–28^.

In a similar study, investigations on direct peptide bond formation using enzymatic activation of the carboxy group on an amino acid followed by nucleophilic substitution was reported. Nakajima *et al*. revealed that aminoacyl-tRNA synthetase enables the catalysis of dipeptide formation via an enzymatically formed aminoacyl-AMP-aminoacyl-tRNA synthetase complex, followed by nucleophilic substitution with amino acids^29^. Dieckmann *et al*. found that *N*-phenylalanyl dipeptides are synthesised by TycA-A using l-phenylalanine and some amino acids (amides)^21^. Our findings are functionally relevant to these reports; however, we also successfully encompassed its applicability beyond peptides to a broad range of general amines.

Previously, Jakubowski had proposed a stimulating enzymatic amide bond formation model involving arginyl-, valyl-, and isoleucyl-tRNA synthetases^30^ (class I aminoacyl-tRNA synthetases), and lysyl-, aspartyl-, and seryl-tRNA synthetases^31^ (class II aminoacyl-tRNA synthetases), catalysing the formation of arginylcysteine, valylcysteine, and isoleucylcysteine and lysylcysteine, aspartylcysteine, and serylcysteine, respectively, in a cysteine-dependent manner. More recently, Abe *et al*. reported a similar enzymatic thiol-based chemistry for amide bond formation using acyl-CoA synthetase^22^ and d-alanine-activating enzyme DltA^23^. In this case, a unique enzymatic adenylation of the substrate and subsequent thioester formation by thiol-containing compounds occurred and the resulting highly reactive thioester bond is immediately rearranged to a more stable amide bond by intramolecular *S*-to-*N* acyl transfer mechanism, similar to the native chemical ligation in chemical protein synthesis^32^. This is an important amide-bond forming methodology; however, it is basically limited to the use of aminoacyl thioester chemistry.

The present amide synthesis method described in this study is advantageous for providing diverse aminoacyl-*N*-alkylamides because of its broader specificity for primary and secondary amines. Importantly, even a d-amino acid was introduced into a dipeptide at the C-terminus position as l-tryptophyl-d-proline. In addition, no extra step is needed, such as CoA addition mediated by phosphopantetheinyl transferase, which is essential to obtain active enzyme of TycA in natural form^33^.

The present method has some advantages in amide synthesis over conventional method. The major organic synthesis commonly relies on *N*-(de)protection and condensation to form amino acid amides. These cumbersome procedures require a protecting group, equal amount of condensation reagent and substrate, and solvent. In contrast, the chemoenzymatic synthesis enables direct ligation of an unprotected amino acid with an amine, which is a straightforward strategy and has greater atom economy. Instead, the chemoenzymatic method requires ATP for amino acid activation. To improve productivity of amide compound synthesis, ATP regeneration should be considered using coupling systems. We are currently investigating ATP regeneration with an adenylation domain platform toward catalytic and practical synthesis of a broad spectrum of amide compounds including peptides, which will be reported elsewhere.

We have attempted to measure the kinetic parameters for general amines; however, quantitation was difficult. First, tryptophyl-*N*-alkylamides, which serves as quantitative standards, were not available from commercial sources. In addition, as shown in stoichiometric analysis (Fig. 4), the decrease in ATP and formation of the amide and AMP did not show any correlation. This is probably due to other possible side reactions, including l-tryptophyl-l-tryptophan formation, caused by competitive substitution by l-tryptophan and unfavourable ATP hydrolysis.

To enhance the reactivity, which may be the rate-limiting factor in the overall reaction, a high concentration (200 mM) of amines was found to be favourable for efficient amide bond formation; however, the reaction velocity remained relatively low even at this concentration in comparison to other enzymatic amide or peptide bond forming reactions. This reduced efficiency is possibly due to the lack of the downstream domains. In the NRPS machinery, the adenylation domain, as well as thiolation domain (also referred to as peptidyl carrier protein domain) and condensation domain are indispensable for peptide bond formation, except for a few minor cases. Their absence resulted in both decreased the rate of amide formation and the broad nucleophile specificity. Similarly, in the case of dipeptide synthesis by aminoacyl-tRNA synthetase, the peptide bond was formed between aminoacyl-AMP and various amino acids; however, the reaction rate is extremely low, suggesting the importance of another conformational factor such as tRNA^29^.

In conclusion, we have revealed a new amide bond formation mechanism involving enzymatic adenylation by TycA-A followed by nucleophilic substitution with primary or secondary amines. Furthermore, TycA-A showed a wide spectrum of substrate flexibility for both mono-substituted tryptophan and d-tryptophan. An improved method for amide bond formation was required, because the current chemical method suffers from low atom economy and results in wastage^34^. The new method demonstrated here would provide a better approach for a more efficient amide bond formation.

## Methods

### Reagents

All chemicals were purchased from Wako Pure Chemical Industries, Ltd. (Osaka, Japan) or Kanto Chemical Co., Inc. (Tokyo, Japan) unless stated otherwise. Biological materials were used as previously described^24^.

### Preparation of TycA-A

The *tycA-A* gene was cloned and overexpressed in *Escherichia coli* as described previously^24^. The supernatant was used for enzyme purification on the Protino Ni-TED 1000 column (Macherey-Nagel, Düren, Germany) equipped on an ÄKTA prime plus (GE Healthcare), and the eluent was desalted on a PD-10 column (GE Healthcare). The enzyme was purified to apparent homogeneity as judged from sodium dodecyl sulfate-polyacrylamide electrophoresis analysis.

### Synthesis of amides by TycA-A

The standard reaction mixture containing 50 mM Tris-HCl buffer (pH 8.0), 10 mM l-tryptophan analogues (acyl donor), 10 mM each of various amines (nucleophile), 20 mM ATP, 20 mM MgSO_4_, and 0.1 mg/mL TycA-A in a total volume of 100 μL was incubated at 37 °C for up to 24 h. The reaction was terminated by heating at 80 °C for 10 min and it was centrifuged at 20,000× *g* for 10 min at 4 °C. To assess stoichiometry, the reaction containing 50 mM Tris-HCl buffer (pH 8.0), 10 mM l-tryptophan analogues (acyl donor), 200 mM l-proline (nucleophile), 5 mM ATP, 5 mM MgSO_4_, and 0.5 mg/mL TycA-A in a total volume of 100 μL was incubated at 37 °C. A small portion of the mixture was withdrawn for the determination of l-tryptophyl-l-proline, ATP, and AMP at each sampling period.

### HPLC analysis

To confirm amide formation, the supernatant was analysed by high performance liquid chromatography (HPLC) equipped with an XTerra MS C18 column (Waters, Milford, MA) kept at 40 °C. The separation was carried out using a linear gradient of methanol (0–100%) in 0.1% (v/v) formic acid solvent for 15 min at a flow rate of 1.0 mL/min, and detection was performed at a wavelength of 214 nm. Whenever it was necessary, the reactants were collected by an HPLC system equipped with a fraction collector (model CHF122SB, Advantec, Tokyo, Japan). ATP and AMP were measured as reported previously^35^.

### Structural analysis of various amides

The MS/MS analysis data were acquired with a Triple TOF4600 mass spectrometer (omics analysis LC-MS system; SCIEX, Framingham, MA) by infusion in the positive modes. The conditions were as follows: ion spray voltage floating, 5.5 kV; declustering potential, 80 V; source temperature, 0 °C; desolvation temperature, 500 °C; ion source gas 2, 20 psi; collision energy, 30 V. The data were collected using Analyst TF 1.6 software (SCIEX). NMR spectra were measured with an AVANCE III spectrometer (Bruker, Billerica, MA). For ^1^H NMR and ^13^C NMR analysis, the compounds dissolved in D_2_O containing trimethylsilyl propanoic acid were used as an internal standard, and spectrometer was operated at 600 MHz and 150 MHz, respectively.

## Electronic supplementary material

Supplementary information
