# Supplementary material for: A chemoenzymatic process for amide bond formation by an adenylating enzyme-mediated mechanism
Source: Sci Rep. 2018 Feb 13;8:2950. doi: 10.1038/s41598-018-21408-8 (PMC5811625; doi:10.1038/s41598-018-21408-8)
Supplement: Supplementary file 1 — Supplementary information [file 41598_2018_21408_MOESM1_ESM.docx]

A chemoenzymatic process for amide bond formation by an adenylating enzyme-mediated mechanism

Ryotaro Hara^1^, Kengo Hirai^2^, Shin Suzuki^2^, Kuniki Kino^1,2*^

^1^Research Institute for Science and Engineering, Waseda University, Tokyo 169-8555, Japan; ^2^Department of Applied Chemistry, Faculty of Science and Engineering, Waseda University, Tokyo 169-8555, Japan.

*Corresponding author: Kuniki Kino (E-mail: kkino@waseda.jp)

Fig. S1. MS/MS fragment spectra of various l-tryptophyl-*N*-alkylamides.

(a) l-tryptophylmethylamine, (b) l-tryptophyldimethylamine, (c) l-tryptophyl-β-alanine, (d) l-tryptophyl-γ-aminobutyric acid, (e) l-tryptophylazetidine, (f) l-tryptophylpyrrolidine, (g) l-tryptophylpiperidine, (h) l-tryptophylazepane, (i) l-tryptophylazocane, (j) l-tryptophyl-d-proline, (k) l-tryptophyl-*cis*-4-hydroxy-l-proline, (l) l-tryptophyl-*cis*-4-hydroxy-d-proline, (m) l-tryptophyl-l-prolinamide, and (n) l-tryptophyl-l-azetidine-2-carboxylic acid.

Fig. S2. NMR spectra of l-tryptophylazetidine. (a) ^1^H NMR and (b) ^13^C NMR.

Fig. S3. NMR spectra of l-tryptophyldimethylamine. (a) ^1^H NMR and (b) ^13^C NMR.

Figure S4. HPLC chromatograms of the reaction mixture containing 6-fluorotryptophan with l-proline (a), dimethylamine (b), and azetidine (c). Closed and open triangles indicate the reaction products and 6-fluorotryptophan, respectively.

Figure S5. HPLC chromatograms of the reaction mixture containing 6-chlorotryptophan with l-proline (a), dimethylamine (b), and azetidine (c). Closed and open triangles indicate the reaction products and 6-chlorotryptophan, respectively.

Figure S6. HPLC chromatograms of the reaction mixture containing 6-bromotryptophan with l-proline (a), dimethylamine (b), and azetidine (c). Closed and open triangles indicate the reaction products and 6-bromotryptophan, respectively.

Figure S7. HPLC chromatograms of the reaction mixture containing 5-hydroxytryptophan with l-proline (a), dimethylamine (b), and azetidine (c). Closed and open triangles indicate the reaction products and 5-hydroxytryptophan, respectively.
